# A rare case of pituicytoma presenting with severe Cushing disease: A case report and review of literature

**DOI:** 10.1097/MD.0000000000017772

**Published:** 2019-11-01

**Authors:** Xiaoman Li, Yang Liu, Yuan Miao, Jinping Wang, Liang Wang, En-Hua Wang

**Affiliations:** a Key Laboratory of Medical Cell Biology, Ministry of Education; b Department of Pathology, College of Basic Medical Sciences and First Affiliated Hospital, China Medical University, Shenyang, China.

**Keywords:** Cushing disease, immunohistochemistry, pituicytoma, pituitary, sellar

## Abstract

**Rationale:**

Pituicytomas are exceptional rare tumors in the sellar and suprasellar regions with clinical manifestations, such as headache, visual disturbance, hypopituitarism, and decreased libido. Unlike that of common pituitary adenoma, the association between pituicytoma and Cushing disease (CD) is extremely rare. There were only 6 reported cases till now. In the current study, we describe an unusual case of pituicytoma associated with severe CD with a recurrence-free follow-up period of 49 months.

**Patient concerns:**

A 32-year-old woman was referred to our hospital with moon face, central obesity, and purple stripes on the lower limbs.

**Diagnoses:**

The plasma cortisol level was 1122 nmol/L. The low-dose dexamethasone suppression test failed to suppress plasma cortisol. This test provided evidence of nonpituitary-dependent CD. However, magnetic resonance imaging demonstrated a sellar mass measuring approximately 7.6 × 5.7 mm. The patient was diagnosed with pituitary microadenoma. Histopathological analysis of the tissue sections based on the findings from the immunohistochemical staining diagnosed it as pituicytoma.

**Interventions:**

Transsphenoidal surgery was performed to remove the pituitary mass.

**Outcomes:**

Within 2 months postoperatively, the patient's blood pressure and cortisol level decreased gradually and normalized on the 6th month when other symptoms of CD also disappeared. The patient is presently free from recurrence 49 months after the initial diagnosis.

**Lessons:**

Based on the postoperative remission, CD was caused by pituitary disorders. A reasonable assumption is that an extremely small coexisting adenoma was not detected by the surgeon and washed out during the dissection. Another possible explanation might be the mass effect caused by this intrasellar lesion.

## 1 Introduction

Pituicytoma, was previously known as “infundibuloma” or “posterior pituitary astrocytoma.”^[1]^ As an extremely rare benign tumor of the sellar and suprasellar regions, it arises from pituicytes of the neurohypophysis and infundibulum.^[2]^ The tumor mass is generally solid, well-circumscribed, and noninfiltrative.^[3]^ According to the location and tumor size, patients with pituicytoma usually exhibit clinical symptoms such as headache, visual disturbance, hypopituitarism, and decreased libido.^[4]^ To date, based on previous studies, the tumor develops mainly in adults rarely in children.^[5–8]^ Radiological findings are nonspecific for pituicytoma; therefore, the most common radiological preoperative diagnosis was pituitary adenoma.^[9,10]^ Currently, with the help of immunohistochemistry (IHC), histological study is still the most accurate examination for this tumor. The best choice of therapy is total resection.

Unlike that of pituitary adenoma, the most common tumor of the sellar, and suprasellar regions, the association between pituicytoma and Cushing disease (CD) is extremely rare. There were only 6 reported cases were reported till now: 4 cases in female adults, 1 in male adult, and 1 in a 7-year-old girl.^[5,6,11–14]^ In the current study, we report a 32-year-old woman with pituicytoma iassociated with severe CD.

## 2 Case report

**Figure 1:**
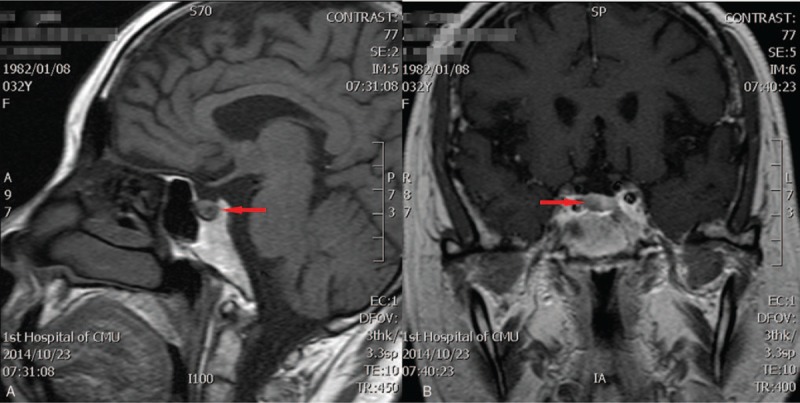
(A) Magnetic resonance imaging (MRI) scan showing a neoplasm (red arrow) that is slightly hypointense on T1-weighted sagittal image. (B) Preoperative coronal MR images.

A 32-year-old woman was referred to our hospital with chief complaint of back pain and kyphosis for 3 years. Three years ago, the patient was diagnosed with “osteoporosis and thoracic compression fracture” at a local hospital. After surgical treatment, the patient did not use regular medication. Last year, the patient experienced fatigue and severe back pain with lower extremity pain after lifting heavy objects, which was not relieved by rest but gradually increased. Upon examination in our clinic, moon face, central obesity, and purple stripes on the lower limbs were noted. The patient was 162 cm in height and weighed 70 kg, with a body mass index of 26.6. The resting blood pressure was 160/100 mm Hg. The patient also had type II diabetes. Laboratory tests showed that lutenizing hormone dramatically decreased to 0.17 mIU/mL; follicle-stimulating hormone level was 2.25 mIU/mL which was within the normal range; aldosterone level was 0.2 ng/mL, plasma renin activity was 4.4 ng/mL, and angiotensin II level was 42.55 pg/mL. Plasma adrenocorticotropic hormone level was within normal range, and plasma cortisol level was 1122 nmol/L (normal <327 nmol/L in the morning). Only slight suppression of plasma cortisol was found during an overnight 8 mg dexamethasone suppression test, from 1291 to 1150 nmol/L. Low-dose dexamethasone suppression test failed to suppress plasma cortisol. This test provided evidence of nonpituitary-dependent CD. However, magnetic resonance imaging demonstrated a sellar mass measuring approximately 7.6 × 5.7 mm (Fig. 1). The lesion was slightly hypointense on T2-weighted images. The patient was diagnosed with pituitary microadenoma. Thus, the diagnosis of pituitary-dependent CD could not be fully ruled out. The controversial test results led to a dilemma in the preoperative diagnosis. Surgeons preferred a diagnosis of pituitary-dependent CD than nonpituitary-dependent CD for this case. After being provided with detailed information, the patient requested to undergo transsphenoidal surgery to remove the pituitary mass. During the surgery, a small tumor was identified and removed. An intraoperative frozen section was examined and diagnosed as pituitary adenoma.

However, after 2 postoperative weeks, plasma cortisol levels and blood pressure did not decrease. Resting blood pressure was 155/98 mm Hg, and plasma cortisol level was 1066 nmol/L. Meanwhile, histopathological of the remaining tissue sections confirmed pituicytoma. The patient refused to undergo further radiotherapy. Within 2 months postoperatively, the patient's blood pressure and plasma cortisol level decreased gradually and normalized on the 6th month when other symptoms of CD also disappeared. The patient is now presently free from recurrence 49 months after the initial diagnosis.

## 3 Pathological findings

**Figure 2:**
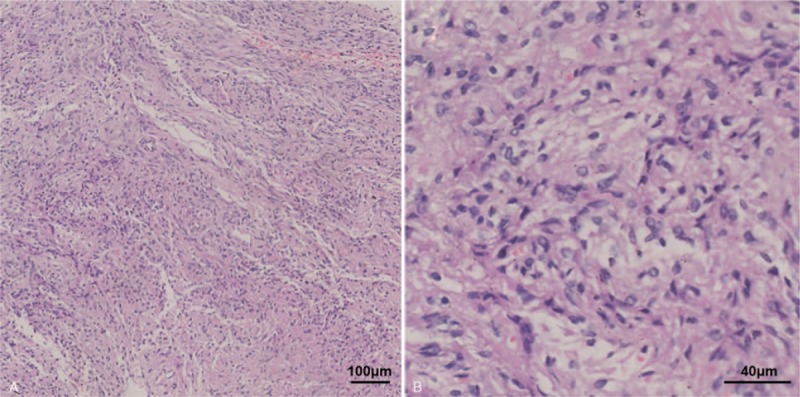
Tumor consisting of interlacing fascicles of elongated, eosinophilic spindle cells, as shown in the hematoxylin and eosin stained sections (A). Nuclei were typically oval to elongated, with slightly irregular borders (B).

**Figure 3:**
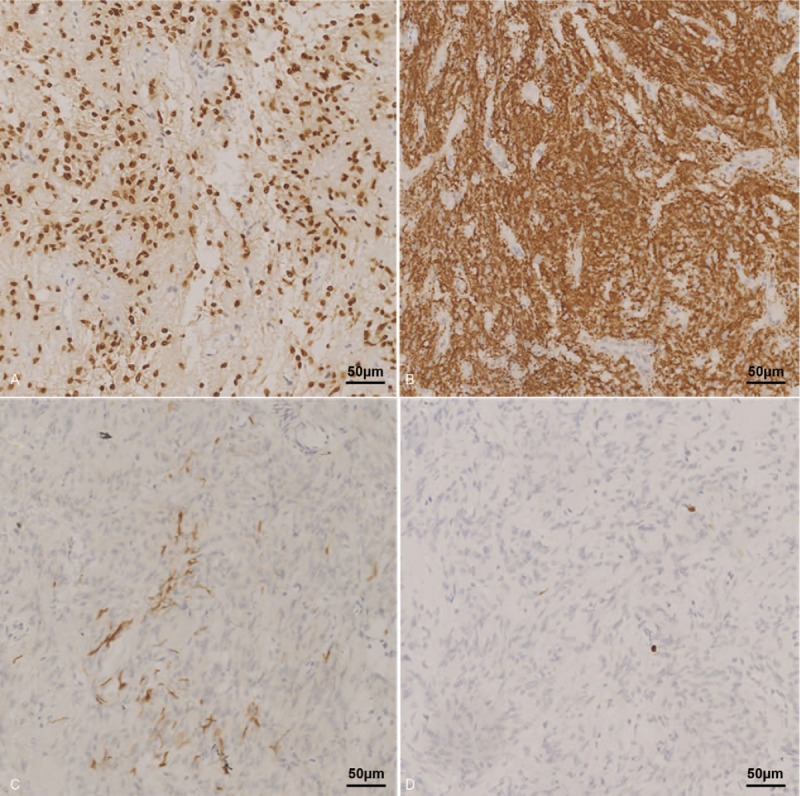
Tumor cells were diffusively positive for TTF-1 (A) and S-100 (B), with focal expression of GFAP (C). The Ki-67 index was <1% (D). GFAP = glial fibrillary acidic protein, TTF-1 = thyroid transcription factor-1.

The surgical specimens consist of multiple small fragments. Most fragments contained 2 parts of tissues: nontumor adenohypophyseal parenchyma and tumor composed of spindle cells, which were disposed in intersecting fascicles and bundles (Fig. 2A). The tumor cells have eosinophilic cytoplasm and showed no nuclear atypia or mitotic activity (Fig. 2B). In the tumor tissue, there were no Rosenthal fibers, granular eosinophilic bodies, or Herring bodies. In tumor cells, diffusive staining of thyroid transcription factor-1 (Fig. 3A), vimentin, and S-100 (Fig. 3B) was assessed by IHC. Glial fibrillary acidic protein was focally stained (Fig. 3C). The tumor cells were negative for epithelial membrane antigen, synaptophysin, periodic acid-Schiff (PAS), and pituitary hormones (adrenocorticotropic hormone, human growth hormone, and prolactin). The Ki-67 index was <1% (Fig. 3D). Nontumor adenohypophyseal parenchyma showed diffuse staining of synaptophysin but was negative for pituitary hormones. Reticulin fibers were intact in nontumor adenohypophyseal parenchyma, suggesting no evident adenoma. With the aid of IHC and PAS staining, other differential diagnoses such as granular cell tumor and pituitary adenomas were excluded.

## 4 Discussion

As an entity by the World Health Organization Classification of Tumours of Endocrine Organs (2017), pituicytoma is a benign spindle cell tumor that originates from pituicytes.^[15]^ To date, there were <80 totally reported cases in English literature. Development in children is rare, in which only 5 cases were reported.^[16]^ All other cases were reported in adults with slightly male predominance.

In most cases, the mass effect of pituicytoma mainly causes visual disturbance, headache, and pituitary insufficiency.^[10,17,18]^ Pituitary-dependent CD was predominantly caused by pituitary adenoma. Cases of patients with pituicytoma associated with pituitary hyperfunction were reported but significantly limited, and those associated with CD were extremely rare. To date, there were only 7 reported cases, including this case, which was associated with CD or Cushingoid features, and one of these cases was reported in a 7-year-old girl.^[5]^ The details of all cases are summarized in Table 1. Among the 6 cases, Chakraborti reported a case of pituicytoma presenting with Cushingoid features and hypertension.^[6]^ Among the other 5 cases with CD, 2 cases were histologically proven to be coexisting with corticotroph hyperplasia or corticotropin-secreting adenoma.^[5,14]^ In 3 cases, no other central nervous lesions were evident besides pituicytoma,^[6,12,13]^ similar to the current case. Then, what would be the cause of remission of CD in this patient? A reasonable assumption is that an extremely small coexisting adenoma was not detected by surgeon and washed out during dissection. Since pituicytomas are commonly quite hemorrhagic during surgery, more precise surgeries and findings are critical to explore small tumor tissues. Another possible explanation might be the mass effect caused by this intrasellar lesion since the compression of the anterior pituitary can also lead to hyperfunctional endocrinological disturbance, including hyperprolactinemia or hypercortisolism, which is similar to that observed in patients with intrasellar craniopharyngioma. In all cases in adults, including the current case, CD was in remission postoperatively, and no recurrence was reported. However, there was no postoperative remission in the case of the 7-year-old girl, and bilateral laparoscopic adrenalectomy was further performed.

**Table 1:**
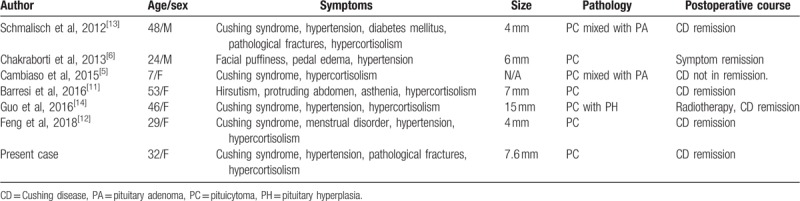
Clinical review of 7 cases published of pituicytomas with CD or Cushingoid features.

In pituicytoma, the radiological studies cannot provide accurate diagnosis in preoperative tests, especially when laboratory test results favor the diagnosis of pituitary adenoma.^[3]^ In the current case, the controversial preoperative test results led to a dilemma for both the surgeon and patient. Based on postoperative remission, CD was caused by pituitary disorders. With the aid of IHC, the tissues sections of pituicytoma can provide the most important evidence in the differential diagnosis of more common pituitary adenoma or other rare pituitary tumors.^[1,19]^ The tumor consisted of spindle-shaped cells arranged in a sheet, storiform patterns, or interlacing fascicles without Rosenthal fibers, eosinophilic granular bodies, or Herring bodies.^[1]^ Nuclear atypia was typically evident. The tumor cells are diffusively positive for vimentin and S-100, focally positive for glial fibrillary acidic protein, and generally negative for epithelial membrane antigen, synaptophysin, chromogranin A, and neurofilament. Unlike pituitary adenomas, pituicytoma tumor cells are negative for pituitary hormones. CD68 and PAS staining were generally negative or variably positive in pituicytoma but positive in granular cell tumor. Another rare pituitary tumor needs to be considered in the differential diagnosis.^[19]^ The Ki-67 index in pituicytoma is usually <2% to 3%. Several studies revealed that pituicytoma is diffusively positive for thyroid transcription factor-1 in the cell nucleus, and this also provides an important clue in differentiating it from pituitary adenoma.^[20]^

Therefore, this report of an extremely rare case demonstrates pituitcytoma in a 32-year-old woman presenting with severe CD. The microscopic features and IHC results support the diagnosis of pituicytoma.

## Acknowledgments

The authors thank Editage (www.editage.cn) for English language editing.

## Author contributions

**Methodology:** Jinping Wang.

**Writing – original draft:** Xiaoman Li, Liang Wang.

**Writing – review & editing:** Yang Liu, Yuan Miao, En-Hua Wang.

Liang Wang orcid: 0000-0003-4015-9385.
